# Assessment of Sensitivity and Specificity of Patient-Collected Lower Nasal Specimens for Sudden Acute Respiratory Syndrome Coronavirus 2 Testing

**DOI:** 10.1001/jamanetworkopen.2020.12005

**Published:** 2020-06-12

**Authors:** Jonathan Altamirano, Prasanthi Govindarajan, Andra L. Blomkalns, Lauren E. Kushner, Bryan Andrew Stevens, Benjamin A. Pinsky, Yvonne Maldonado

**Affiliations:** 1 Department of Pediatrics, Stanford University School of Medicine, Stanford, California; 2 Department of Emergency Medicine, Stanford University School of Medicine, Stanford, California; 3 Department of Pathology, Stanford University School of Medicine, Stanford, California

## Abstract

This prognostic study compares the diagnostic equivalence of patient-collected lower nasal specimens with that of health care worker–collected oropharyngeal specimens for sudden acute respiratory syndrome coronavirus 2 (SARS-CoV-2) testing.

## Introduction

Since the emergence of the sudden acute respiratory syndrome coronavirus 2 (SARS-CoV-2) outbreak in Wuhan, China, in December 2019, the virus has spread to 173 countries, resulting in 3 855 788 confirmed cases and 265 862 deaths as of May 9, 2020.^1^ Stanford Health Care was one of the first nonfederal facilities to obtain US Food and Drug Administration approval for a proprietary test using reverse transcriptase–polymerase chain reaction for SARS-CoV-2 using nasopharyngeal and oropharyngeal specimens on March 2, 2020. However, specimen collection must be done by health care workers and requires extensive use of personal protective equipment. To minimize the risk of exposure during testing, reduce personal protective equipment use, and increase access to testing, we compared the diagnostic equivalence of a modified specimen collection method, patient-collected lower nasal swabs, with that of the current clinical standard, health care worker–collected oropharyngeal swabs. If the 2 methods proved to be diagnostically equivalent, patients would be able to collect specimens themselves without exposing health care workers to respiratory secretions.

## Methods

This prognostic study was approved by the Stanford University institutional review board. Participants provided oral informed consent to clinical research coordinators and then signed a consent form with the physician who collected the oropharyngeal swab. This study complies with the Standards for Reporting of Diagnostic Accuracy (STARD) reporting guideline.

The target population was Stanford Health Care outpatients with a reverse transcriptase–polymerase chain reaction test that was positive for SARS-CoV-2 in March 2020. We included a convenience sample of patients who consented to be contacted by our study staff. Our study staff obtained informed consent remotely, to minimize exposure of research staff to infected patients and to reduce the duration of study visits, and sent instructional materials electronically. Health care workers were excluded because of their familiarity with specimen collection, as were patients enrolled in drug trials for SARS-CoV-2.

After informed consent was obtained, participants were scheduled to return to Stanford Health Care for drive-through collection of 3 specimens using a patient-collected lower nasal swab (Puritan Sterile Foam Tipped Applicator; Puritan Medical Products), a physician-collected lower nasal swab (Puritan Sterile Foam Tipped Applicator; Puritan Medical Products), and a physician-collected oropharyngeal swab (FLOQ Swabs; Copan Diagnostics). During the visit, participants received a $20 incentive. The 3 specimens were placed in separate 3-mL tubes of viral transport medium (M4RT Transport; Remel MicroTest), stored in a cooler, and delivered to the Stanford Clinical Virology Laboratory. Specimens were tested for SARS-CoV-2 using reverse transcriptase–polymerase chain reaction targeting the envelope or open reading frame 1 ab genes.^2,3^

We report participant demographic characteristics, sensitivity and specificity, and discordant probabilities with 95% binomial CIs of patient-collected lower nasal compared with physician-collected oropharyngeal specimens. All analyses were performed with SAS statistical software version 9.4 (SAS Institute). Data analysis was performed in May 2020.

## Results

Of 129 eligible SARS-CoV-2–infected patients, 30 participated in the study (mean [SD] age, 48.2 [16.0] years; 16 men [53%]). We do not have reasons for refusal for nonparticipants. Participants were predominantly white (20 participants [66%]), with no sex or age group predominance. Twelve participants (40%) self-reported possible exposure to SARS-CoV-2. Cough (20 participants [67%]), fever (13 participants [43%]), and sore throat (8 participants [27%]) were commonly reported symptoms during the first visit. Approximately one-half of the participants had documentation of chronic medical conditions (16 participants [53%]), and 5 participants (17%) tested positive for coinfections with other respiratory viruses (Table 1).

**Table 1.** Demographic Characteristics of Participants With a Positive Sudden Acute Respiratory Syndrome Coronavirus 2 Diagnosis in Stanford, California, March 2020

Characteristic	Participants, No. (%)
Sex	
Male	16 (53)
Female	14 (47)
Race/ethnicity	
White	20 (66)
Asian	3 (10)
Hispanic or Latino	2 (7)
Native Hawaiian or Pacific Islander	1 (3)
Other	2 (7)
Unknown	2 (7)
Age, y	
19-30	3 (10)
31-40	8 (27)
41-50	5 (17)
51-60	7 (23)
61-70	3 (10)
71-80	4 (13)
Possible exposure	
Travel	5 (17)
Close contact with a patient with coronavirus disease 2019	4 (13)
Close contact with influenza-like illness	3 (10)
Exposure unknown	18 (60)
Symptoms at initial visit	
Cough	20 (67)
Fever	13 (43)
Sore throat	8 (27)
Shortness of breath	7 (23)
Congestion	6 (20)
Myalgia	6 (20)
Chest pain	5 (17)
Headache	4 (13)
Rhinorrhea	3 (10)
Diarrhea	2 (7)
Fatigue	2 (7)
Weakness	1 (3)
Decreased sense of taste	1 (3)
Syncope	1 (3)
Comorbidities	
Any comorbidity	16 (53)
Hypertension	5 (17)
Hyperlipidemia	5 (17)
Asthma	3 (10)
Diabetes	2 (7)
Obesity (body mass index >30)^a^	2 (7)
Other^b^	11 (37)
Coinfections	
Any coinfections	5 (17)
Rhinovirus	1 (3)
Respiratory syncytial virus	1 (3)
Seasonal coronavirus	1 (3)
Parainfluenza (1, 3, or 4)	2 (7)

^a^ Body mass index is calculated as weight in kilograms divided by height in meters squared.

^b^ Includes comorbidities such as chronic pain, arthritis, anxiety, depression, and constipation.

We observed diagnostic equivalence across the 3 methods of specimen collection (Table 2). Eleven participants (37%) had test results that were positive for SARS-CoV-2 across patient- and physician-collected specimens, and 18 participants (60%) had results that were negative for SARS-CoV-2 across patient- and physician-collected specimens. The only discordant result was a participant whose self-collected nasal specimen tested positive, whereas both of their physician-collected specimens tested negative (3.30%; 95% CI, 0.08%-17.00%). The sensitivity of the patient-collected specimens was 100% (95% CI, 72%-100%), and the specificity was 95% (95% CI, 74%-100%).

**Table 2.** Detection of Sudden Acute Respiratory Syndrome Coronavirus 2 by Sampling Method and Days from Symptom Onset to Study Sample Collection in Stanford, California, March 2020

Participant	Time from self-reported symptom onset, d	Self-collected nasal swab	Physician-collected nasal swab	Physician-collected oropharyngeal swab
1	18	Detected	Detected	Detected
2	10	Not detected	Not detected	Not detected
3	8	Detected	Detected	Detected
4	13	Detected	Detected	Detected
5	22	Not detected	Not detected	Not detected
6	9	Not detected	Not detected	Not detected
7	NA^a^	Not detected	Not detected	Not detected
8	26	Not detected	Not detected	Not detected
9	28	Detected	Detected	Detected
10	9	Detected	Detected	Detected
11	8	Detected	Detected	Detected
12^b^	NA^a^	Detected	Not detected	Not detected
13	16	Not detected	Not detected	Not detected
14	14	Not detected	Not detected	Not detected
15	NA^a^	Not detected	Not detected	Not detected
16	10	Not detected	Not detected	Not detected
17	37	Not detected	Not detected	Not detected
18	24	Not detected	Not detected	Not detected
19	NA^a^	Detected	Detected	Detected
20	16	Not detected	Not detected	Not detected
21	9	Not detected	Not detected	Not detected
22	NA^a^	Not detected	Not detected	Not detected
23	21	Not detected	Not detected	Not detected
24	15	Not detected	Not detected	Not detected
25	23	Not detected	Not detected	Not detected
26	10	Detected	Detected	Detected
27^c^	4	Detected	Detected	Detected
28	NA^a^	Not detected	Not detected	Not detected
29	10	Detected	Detected	Detected
30	NA^a^	Detected	Detected	Detected

Abbreviation: NA, not applicable.

^a^ Symptom onset was not recorded in the electronic medical record for participants 7, 12, 15, 19, 22, 28, and 30.

^b^ Participant 12 presented with discordant results, with a positive result for the self-collected nasal sample but negative results from both physician-collected samples.

^c^ Participant 27 was asymptomatic but had been exposed to sudden acute respiratory syndrome coronavirus 2 by close contact 4 days before the study swab was obtained.

## Discussion

These findings contribute to the recently released US Food and Drug Administration guidance^4^ that lists patient-collected lower nasal swab as an acceptable specimen collection method for SARS-CoV-2 testing. Self-collected lower nasal swabs could also be used for home- or office-based testing of asymptomatic patients. However, these preliminary findings are limited by small sample size, have limited generalizability, and need to be validated further in diverse clinical settings. These validation efforts are currently under way at our institution.
