# Error in Title, Text, and Tables

**DOI:** 10.1001/jamanetworkopen.2020.14910

**Published:** 2020-07-13

In the Research Letter titled “Assessment of Sensitivity and Specificity of Patient-Collected Lower Nasal Specimens for Sudden Acute Respiratory Syndrome Coronavirus 2 Testing,”^1^ published June 12, 2020, in the title, text, and tables, the term *sudden acute respiratory syndrome coronavirus 2* should be *severe acute respiratory syndrome coronavirus 2*. In addition, the corresponding author’s address has been corrected. This article has been corrected.^1^
